# A Unit Compound Structure Design: Poisson’s Ratio Is Autonomously Adjustable from Negative to Positive

**DOI:** 10.3390/ma16051808

**Published:** 2023-02-22

**Authors:** Guanxiao Zhao, Tao Fu

**Affiliations:** Faculty of Mechanical and Electrical Engineering, Kunming University of Science and Technology, Kunming 650093, China

**Keywords:** shape memory polymer, multi-cell, Poisson’s ratio, metamaterial, bidirectional memory effect, auxetic

## Abstract

The shape memory polymer (SMP) is a new type of smart material that can produce a shape memory effect through the stimulation of the external environment. In this article, the viscoelastic constitutive theory of the shape memory polymer and the mechanism of the bidirectional memory effect of the shape memory polymer are described. A chiral poly cellular circular concave auxetic structure based on a shape memory polymer made of epoxy resin is designed. Two structural parameters, α and β, are defined, and the change rule of Poisson’s ratio under different structural parameters is verified by ABAQUS. Then, two elastic scaffolds are designed to assist a new type of cellular structure made of a shape memory polymer to autonomously adjust bidirectional memory under the stimulation of the external temperature, and two processes of bidirectional memory are simulated using ABAQUS. Finally, when a shape memory polymer structure implements the bidirectional deformation programming process, a conclusion is drawn that changing the ratio β of oblique ligament and ring radius has a better effect than changing the angle α of oblique ligament and horizontal in achieving the autonomously adjustable bidirectional memory effect of the composite structure. In summary, through the combination of the new cell and the bidirectional deformation principle, the autonomous bidirectional deformation of the new cell is achieved. The research can be used in reconfigurable structures, tuning symmetry, and chirality. The adjusted Poisson’s ratio achieved by the stimulation of the external environment can be used in active acoustic metamaterials, deployable devices, and biomedical devices. Meanwhile, this work provides a very meaningful reference for the potential application value of metamaterials.

## 1. Introduction

**Figure 1:**
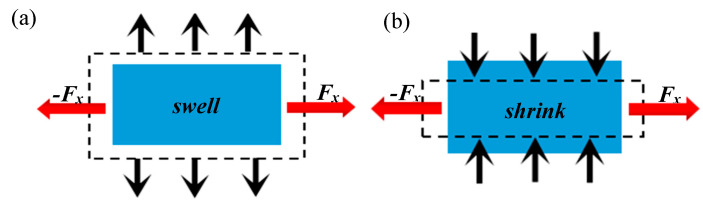
(**a**) Auxetic structure. (**b**) Conventional structure.

The mechanical metamaterial of an auxetic structure has different characteristics than conventional materials because of the auxetic effect, as shown in Figure 1. Liu et al. [1] used the finite element method to compare the concave hexagonal honeycomb with the conventional hexagonal honeycomb. The results clearly show that the mechanical properties of the structure with an auxetic effect are much better than those of the conventional structure. In the 1980s, Lakes [2] put ordinary polyurethane foam into aluminum molds. After a series of heat and cooling treatments, he obtained polyurethane foam with a Poisson’s ratio of −0.7. Since then, research has gradually developed in this field. In recent years, through the continuous exploration and research of researchers, a large number of single-cell auxetic structures have been designed, such as the concave polygon structure [3], rotating rigid body structure [4], chiral structure [5], and perforated plate structure [6]. Based on these auxetic structures, researchers have made significant improvements to these structures, and they found that they could have better mechanical properties by changing the original structure. Li et al. [7] designed a new structure having a sine-wave shape with a negative Poisson’s ratio effect based on the concave hexagonal unicellular structure. The Poisson’s ratio could reach −0.58 and −1.12 by adjusting the period of the sine-wave shape at the right ratio, each topological structure having a different ability to absorb energy. Li et al. [8] designed a hollow-element auxetic structure that could adjust its Poisson’s ratio via geometric variables without changing the parent structure. The Poisson’s ratio of this structure could be adjusted between 0.5 and −1. Galt et al. [9] designed a new type of structure with a negative Poisson’s ratio based on rotating rigid elements using multiple levels of the same unit. Then, they compared Poisson’s ratio at different levels; this idea of controlling the performance of the whole structure system by changing the structure level improved the generality of the parent structure. Lim et al. [10] summarized a large number of auxetic structures and analyzed the relationship between these structures in detail, which provided an idea for the design of new auxetic structures. In conclusion, with the exploration and deep understanding of negative–Poisson’s ratio structural materials, they have been widely used in various fields, including engineering construction [11], biomedical domain [12], aerospace [13], and textiles [14].

Shape memory material is a smart material that can sense the stimulation of external environmental changes and adjust the mechanical parameters. The materials with shape memory effects include shape memory alloys (SMAs), shape memory polymers, and shape memory ceramics (SMCs). Among them, the shape memory polymer has excellent large deformation recovery performance. Compared with a shape memory alloy, whose recovery strain is only 8%, the recovery rate of a shape memory polymer can exceed 400% [15]. The first shape memory polymer was developed in 1984 by Cdf Chime in France, and the shape memory effect was discovered by Jaeon in Japan. Shape memory polymers can sense and respond to the external environment, which includes physical electrical stimulation, thermal stimulation, light stimulation, and chemical stimulation (by changing the pH value [16]). Among these methods, thermal stimulation is commonly used to achieve the shape memory effect. In addition to the excellent recovery ability, a shape memory polymer has the characteristics of easy degradation [17] and easy processing. It is also widely used in aerospace [18], biomedical devices [19], and textile manufacturing [20].

It is worth noting that shape memory polymers can combine with different physical structures to achieve better programmability because of their great response rates. So far, there are relatively few examples of combining auxetic structural with smart materials to achieve auxetic structural metamaterial programmability. Early on, magnetic control was used, in which an elastomer embedded with magnetic particles is placed in a controlled magnetic field to achieve the programmability of physical structures [21]. However, the method of using magnetic field control to achieve structure programmability lacks a locking ability, and once the controlled magnetic field is removed, the deformed structure returns to its original shape. Therefore, more researchers have been trying to increase the stability of structural deformation. Wei et al. [22] designed a unique negative Poisson’s ratio structure, and the relationships among the elastic modulus, Poisson’s ratio, and lattice structure parameters of the structure were studied using theoretical and finite element methods. Then, they combined the structure with a shape memory polymer to achieve programmability. He et al. [23] simulated the transformation mode of a periodic circular cell based on a shape memory polymer using the finite element method. Then, the viscoelastic properties of the shape memory polymer in the shape memory period were explained by the time–temperature superposition principle. The influence of nominal strain rate and temperature on the shape memory period was obtained using the finite element method, and they found that the structure revealed an auxetic effect in the process of deformation. Li et al. [24] designed a quadrilateral structure composed of two kinds of materials. Through the difference in the thermal expansion coefficient of the material, the Poisson’s ratio of the material could be adjusted according to the temperature.

Based on the above research, a chiral multicellular circular concave structure is designed in this article. The Poisson’s ratio can change from positive to negative by changing the structural parameters of the structure during the stretching process, which is verified using finite element software. Combining a shape memory polymer with an elastic scaffold, a composite structure that can achieve bidirectional deformation according to the temperature variation is designed, and its Poisson’s ratio can be converted between positive and negative. Although the previous related articles could achieve the conversion of Poisson’s ratio through shape memory materials, most changes were in the negative range of Poisson’s ratio, and the deformation was unidirectional deformation. After the shape returns to its initial form, it needs to be controlled by an external force. The control process is complex, and the flexible transformation between positive and negative Poisson’s ratio cannot be achieved. The composite structure benefits from the principle of bidirectional deformation and the advantages of the new structure, which can achieve the flexible transformation of the Poisson’s ratio from positive to negative or from negative to positive simply via external temperature stimulus. Because this kind of concave composite structure has a unique ring-ligament configuration, it may have better impact resistance than ordinary concave hexagonal cells under a negative Poisson’s ratio. The design of such composite structures with bidirectional flexible transitions of positive and negative Poisson’s ratios provides a reference for the potential application fields of deformable metamaterials.

## 2. Viscoelastic Theory of Shape Memory Polymers

**Figure 2:**
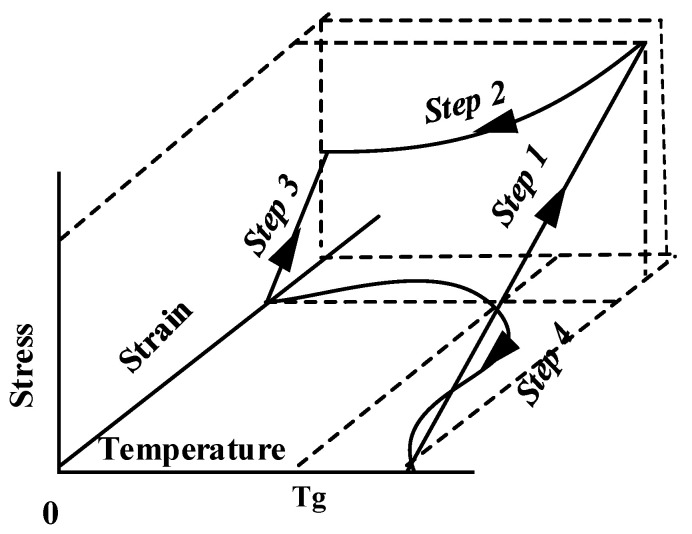
Stress, strain, and temperature curves of shape memory polymer.

The shape memory polymer has two states: rubber state and glassy state. The shape memory polymer reaches the rubber state when heated to a certain temperature (T_g_), and then when it cools to below the T_g_ temperature, it returns to the glassy state. The deformation of a shape memory polymer can be controlled with a heat source, and the shape memory effect is generated in this process. The relationship between temperature, stress, and strain is shown in Figure 2 [25].

**Figure 3:**
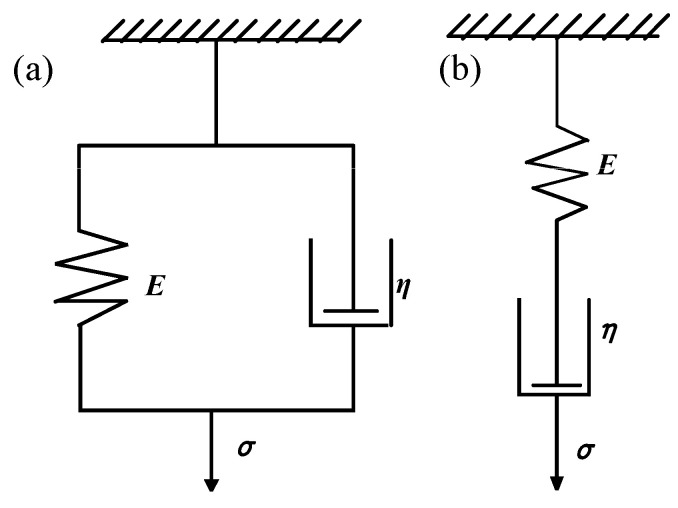
(**a**) Voigt–Kelvin parallel model. (**b**) Maxwell series model [26].

The mechanical response of the shape memory polymer is related to ambient temperature, loading history, loading time, etc., as shown in Figure 2. The material is in a glassy state when the temperature of the shape memory polymer is below T_g_, and it can be regarded as a linear elastic material. When the material is above the temperature T_g_, the modulus of the material decreases rapidly, showing a rubbery state. However, the mechanical properties of the shape memory polymer are between elastic and viscous in the rubbery state. The material cannot be described using the ideal spring model or ideal sticky pot model in the rubber state. In order to better describe the material properties, a more reasonable constitutive model can be obtained by combining the spring and the sticky pot, as shown in Figure 3. Its constitutive relation is as follows:

(1) $$\dot{\varepsilon }=\frac{\dot{\sigma }}{E}+\frac{\sigma }{\eta }$$

where $\dot{\varepsilon }$ is the derivative of strain with respect to time, σ is external stress, η is the viscosity of the sticking pot, and E is the material’s Young’s modulus.

**Figure 4:**
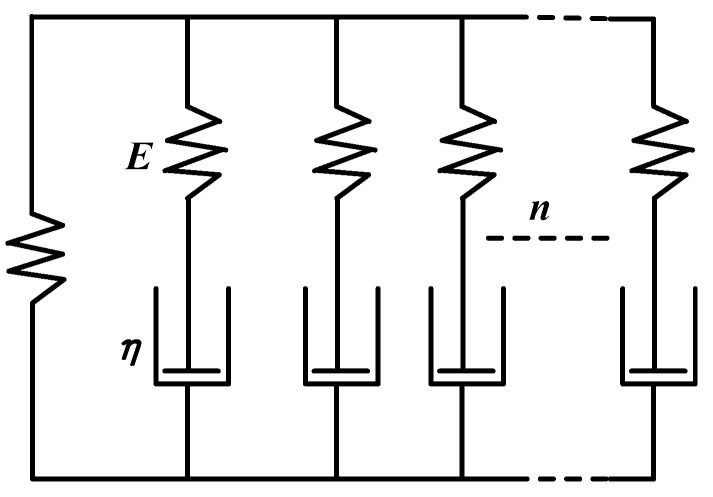
Generalized Maxwell model.

However, while the Maxwell series model can describe the typical stress relaxation behavior, it cannot describe the creep behavior. The Voigt–Kelvin parallel model can describe the creep behavior but not the stress relaxation behavior. Combining the advantages of the two models, a generalized Maxwell model is used to simulate viscoelasticity by fitting the WTF function [23], as shown in Figure 4.

The constitutive relation is expressed as follows:

(2) $$\sigma (t)=\int_{0}^{t}2G_{1}(t)\frac{\partial }{\partial_{\tau }}e(\tau )d\tau$$

The relaxation modulus of viscoelastic materials is described by the Prony series as follows:

(3) $$G_{i}(t)=G_{0}\sum_{i=1}^{n}w_{i}e^{\left(-\frac{t}{\tau_{i}}\right)}$$

where σ(t) is stress as a function of time, $G_{i}(t)$ is the shear modulus relaxation function, $G_{0}$ is the initial shear modulus, n is the number of Maxwell units, $\tau_{i}$ is the relaxation time of Maxwell units, $\tau_{i}=\frac{\eta_{i}}{G_{i}}$, and $w_{i}$ is the weighting factor.

The basic genetic integral formula of linear elastic isotropic viscoelasticity is

(4) $$\sigma (t)=\int_{0}^{t}2G(\tau -\tau \prime )\frac{\partial }{\partial_{\tau }}\dot{e}dt\prime +I\int_{0}^{t}K(\tau -\tau \prime )\dot{\phi }dt\prime$$

where e is the mechanical deviation strain, ϕ is the volumetric strain, I is the strain invariant, and G is the shear modulus. Both ***I*** and *G* are functions of the relaxation time $\tau_{i}$.

Integrate the genetic formula using integration by parts:

(5) $$\sigma (t)=2G_{g}e(t)+\int_{0}^{\tau }2\dot{G}(\tau \prime )e(t-t\prime )+I(K_{g}\phi (t)+\int_{0}^{\tau }\dot{K}(\tau \prime )\phi (t-t\prime )d\tau \prime )$$

where $G_{g}$ and $K_{g}$ are the initial shear modulus and bulk modulus, respectively.

In an environment with varying temperatures, the relaxation time τ is a function of temperature, and the functional form can be represented as follows:

(6) $$\frac{d\tau }{dt}=\frac{1}{a_{T}(T(t))}$$

where $a_{T}(T)$ is the shift factor of the WLF equation [26]. The expression can be expressed as follows:

(7) $$\mathrm{lg}(a_{T})=\frac{-C_{1}(T-T_{ref})}{C_{2}+T-T_{ref}}$$

where $C_{1}$ and $C_{2}$ are parameters of the material itself and $T_{ref}$ is the reference parameter in the WLF equation, defined as the critical temperature for the transition between the glass state and rubber state of the shape memory polymer in the finite element simulation. According to the experiment by Diani et al. [26], we apply the parameters of $C_{1}=10.17$, $C_{2}=47.35$, and $T_{ref}=50$ °C in the WLF equation.

In the integral of the genetic formula, G(t) and K(t) can be represented by Prony functions:

(8) $$G(\tau )=G_{i}+\sum_{i=1}^{n_{g}}g_{i}G_{0}e^{\frac{-\tau }{\tau_{i}}}$$

(9) $$K(\tau )=K_{i}+\sum_{i=1}^{n_{k}}k_{i}e^{\frac{-\tau }{\tau_{i}}}$$

where $G_{i}$ is the shear modulus at time i, $K_{i}$ is the bulk modulus at time i, $g_{i}$ and $k_{i}$ are the simulation parameters regarding $G_{i}$ and $K_{i}$ in ABAQUS. In addition, Prony is the normalization parameter in ABAQUS; when the bulk modulus is constant, $k_{i}=0$, and $n_{g}=n_{k}=n$ is the number of terms in ABAQUS.

The elastic behavior of the material is represented by the neo-Hookean model hyperelasticity behavior:

(10) $$U=C_{10}({\bar{I}}_{1}-3)+\frac{1}{D_{1}}{\left(J_{el}-1\right)}^{2}$$

(11) $$C_{10}=\frac{G_{0}}{2}D_{1}=\frac{2}{K_{0}}$$

where $G_{0}$ is the initial shear modulus, $K_{0}$ is the initial bulk modulus, $I_{1}$ is the first strain invariant, and $J_{el}$ is the elastic volumetric strain.

(12) $$J_{el}=\frac{J}{J_{th}}$$

(13) $$J_{th}={\left(1+\varepsilon_{th}\right)}^{3}$$

where J is the total strain, $J_{th}$ is the theoretical dependent variable, and $\varepsilon_{th}$ is the linear expansion thermal strain. The parameters of hyperelasticity, viscoelasticity, and expansion can be entered directly in ABAQUS. All the parameters and derivation work in this section are presented in articles by Diani et al. [27,28].

## 3. Bidirectional Shape Memory Effect Theory Based on SMP Composite Structure

**Figure 5:**
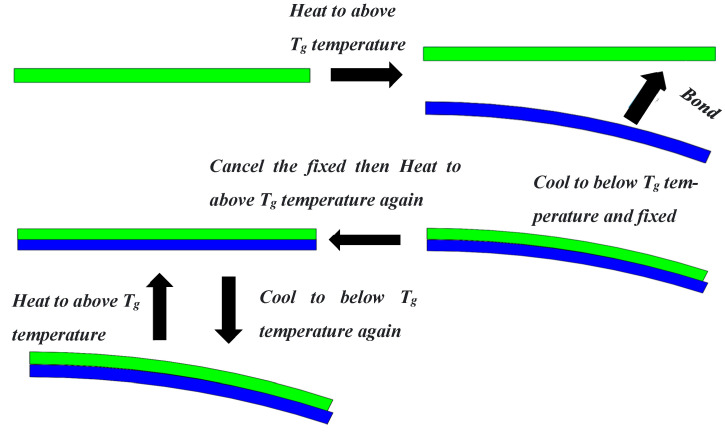
Principle of bidirectional deformation.

Although shape memory polymers have higher deformation recovery rates than shape memory alloys, the shape memory polymers lack the ability of bidirectional deformation, and they cannot deform bidirectionally like shape memory alloys. Most articles about the combination of shape memory polymers and auxetic structures employ unidirectional memory. There was no way to achieve a true sense of bidirectional regulation. After returning to the original state, manual control is needed in order to achieve the desired state. In this section, we use a special elastic scaffold to assist in shape memory polymer deformation. Firstly, the structure made from the shape memory polymer is heated to a temperature above the glassy state temperature (T_g_). The shape memory polymer material can enter the rubbery state when the temperature is above T_g_, and Young’s modulus is several orders of magnitude different from that of the elastic scaffold. At this point, bond the rubbery shape memory polymer structure to a specially prepared elastomer. After bonding, fix the composite structure composed of elastomer and shape memory polymer structure. Then, cool the composite structure to below the temperature T_g_, and the shape memory polymer structure changes back to the glass state after cooling. After cooling, we can remove the previous fixation. Now, the state of the composite structure is the second state that we want to obtain. Then, we need to reheat the composite structure above T_g_; at this time, the shape memory polymer structure produces a shape memory effect, owing to the increase in temperature. The shape memory polymer structure drives the entire composite structure back to its original state through its shape memory effect. At this point, the elastomer has a prestress due to the shape memory polymer structure. Then, cool the temperature of the composite structure again. When the temperature drops below T_g_, this elastomer drives the whole composite structure to restore to the second state (because there is no fixation and the composite structure elastomer contains prestress). Then, if we reheat the whole composite structure, the shape memory polymer can drive it back to its original state because of the shape memory effect. By repeatedly heating and cooling to certain temperatures, we can obtain the shape memory polymer composite structure with two-way deformation. The deformation principle is shown in Figure 5.

## 4. Bidirectional Shape Memory Effect Theory Based on SMP Composite Structure

**Figure 6:**
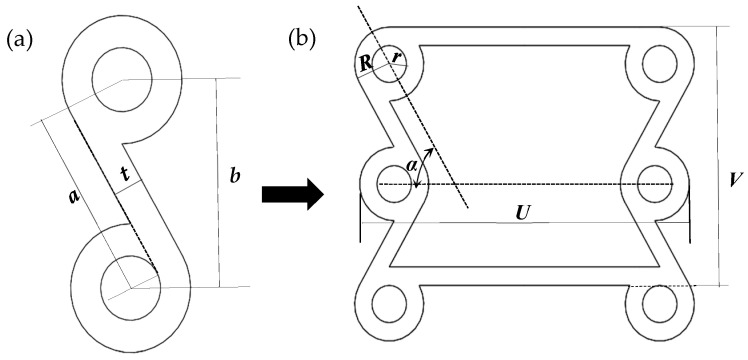
(**a**) The smallest unit of a cell. (**b**) Chiral multicellular circular concave structure.

The concave structure of the chiral multicellular circle is shown in Figure 6. The single-cell structure is composed of rings and ligaments.

In Figure 6a,b, α is the angle between the oblique ligament and the horizontal ligament. The inner diameter of the ring is r, and the outer radius of the circle is R. The thickness of the ligament is t. Because the ligament thickness has little effect on the overall Poisson’s ratio *v*, we can ignore this thickness. The length of the oblique ligament is *a*, and the vertical distance between the centers of the two rings is *b*. The relationship between these structural parameters should satisfy $\arcsin\frac{b}{a}=\alpha$.

The mechanical properties of the single-cell structure will change obviously when any parameter is changed. The valid change parameters are α (the angle between the oblique ligament and the horizontal ligament) and β (the ratio between the radius of the ring and the ligament). We define the displacement in the Y direction as Δy and the displacement in the X direction as Δx. Poisson’s ratio v is $-\frac{\Delta x/U}{\Delta y/V}$.

**Figure 7:**
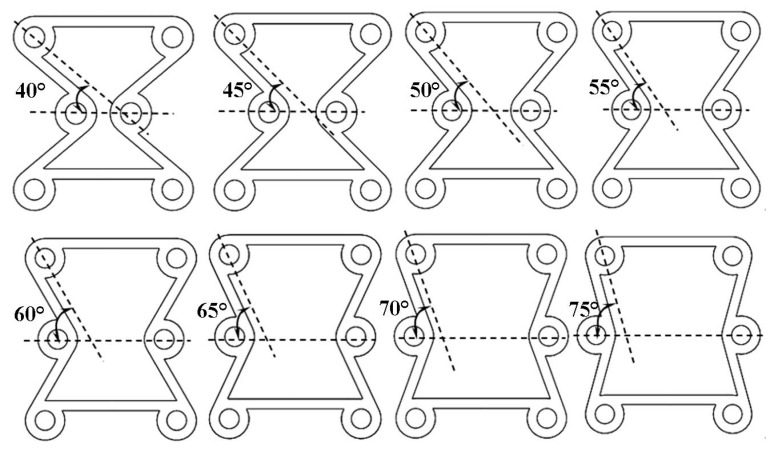
At β = 1:3, the cells take different angles α of 40°–75°.

The ratio β between the ligament and the radius of the ring is 1:4. Keep the β constant, and change the parameters of α. The parameters of α are respectively defined as 40°, 45°, 50°, 55°, 60°, 65°, 70°, and 75°. The outer diameter (R) of the ring is defined as 10 mm, as shown in Figure 7.

**Figure 8:**
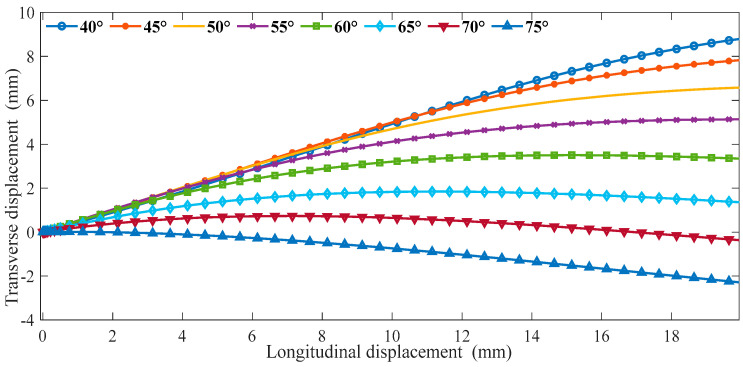
The relationship between transverse and longitudinal displacements (Fix β).

**Figure 9:**
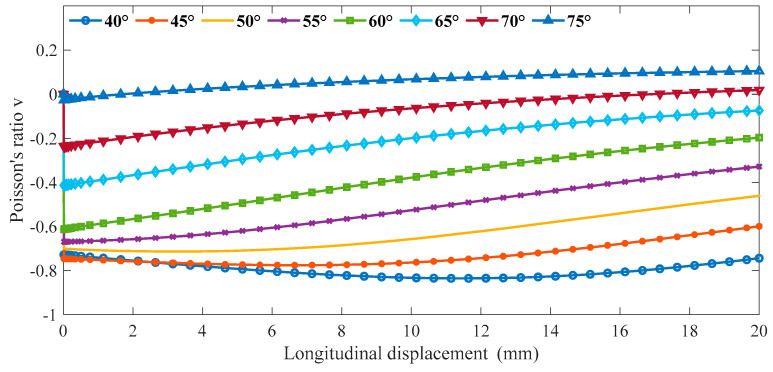
The relationship between longitudinal displacements and Poisson’s ratio *v* (Fix β).

We input cells with different α into ABAQUS for the finite element analysis. The material properties define the shape memory polymer based on the epoxy resin in Section 2, and the material parameters use the data in Appendix A. The models use an eight-node linear hexahedral element (C3D8R). The reduction integral algorithm and hourglass control are adopted. Fix the bottom ligament, and a displacement of 20 mm is applied to the structure along the Y in ABAQUS. We can obtain the following data between longitudinal displacement and Poisson’s ratio *v*. As shown in Figure 8, as α increases gradually, the lateral displacement decreases gradually when the same longitudinal displacement occurs in the cell. In Figure 9, the Poisson’s ratio shows an increasing trend with the gradual increase of α. In the variable range of the structure, the maximum Poisson’s ratio can reach about 0, and the smallest Poisson’s ratio is around 0.8.

In the following, we discuss the influence of two parameters on Poisson’s ratio *v*. We set the angle α to some certain parameter and change the ratio β between the radius of the ring and the oblique ligament. The outer diameter (R) of the ring is defined as 10 mm.

**Figure 10:**
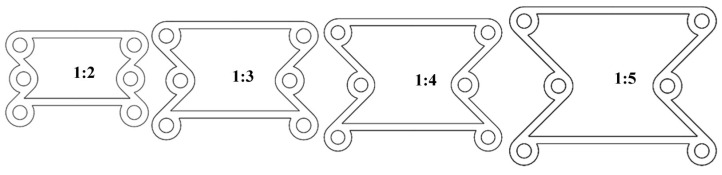
Keeping the α = 45° constant and changing the β to 1:2–1:5.

When the angle α=45°, the ratio β between the radius of the ring and the ligament is defined as 1:2, 1:3, 1:4, and 1:5, as shown in Figure 10.

**Figure 11:**
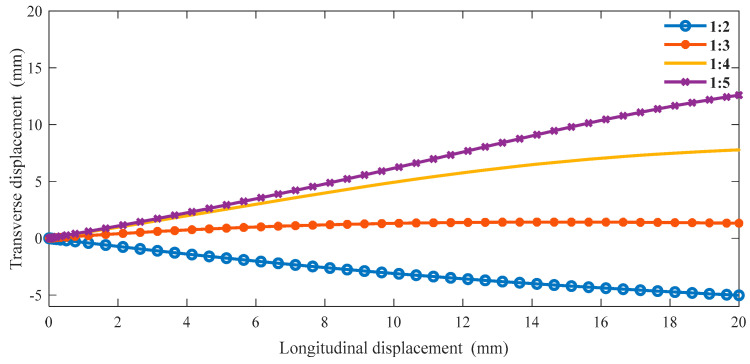
The relationship between transverse and longitudinal displacements under different β (α = 45°).

**Figure 12:**
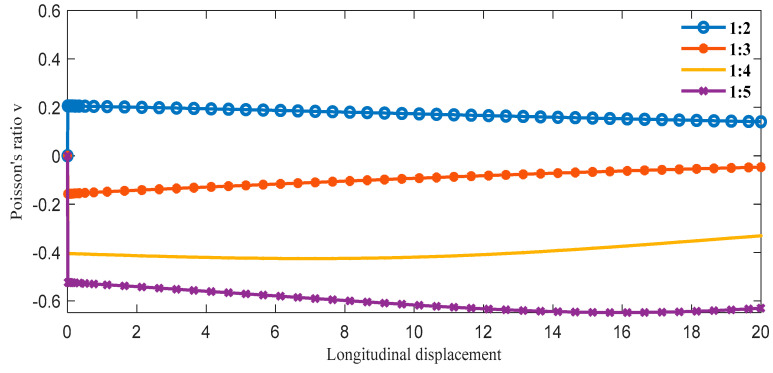
The relationship between longitudinal displacements and Poisson’s ratio *v* under different β (α = 45°).

The boundary conditions are the same as in the previous section in ABAQUS, and we can obtain the following data. From Figure 11, as the proportion of the oblique ligament length in β gradually increases, the lateral displacement shows an upward trend under the same longitudinal displacement of the cell. Additionally, when β is greater than 1:4, the slope is negative, and when β is less than 1:3, the slope is positive. In Figure 12, when β is less than 1:3, the Poisson’s ratio of the structure is very close to 0 or greater than 0. In the variable range of the structure, the maximum Poisson’s ratio can reach up to 0.2, and the smallest Poisson’s ratio is around 0.6.

**Figure 13:**
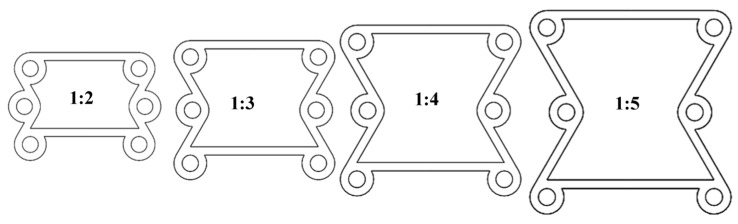
Keeping α = 60° and changing β from 1:2–1:5.

When α=60°, the ratio β between the radius of the ring and the ligament is set to 1:2, 1:3, 1:4, and 1:5, as shown in Figure 13.

**Figure 14:**
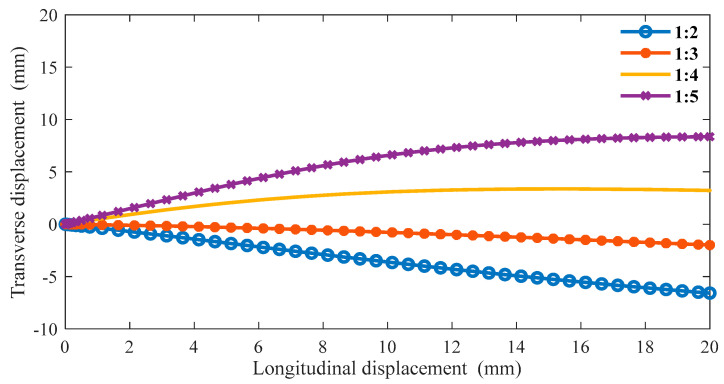
The relationship between transverse and longitudinal displacements under different β (α = 60°).

**Figure 15:**
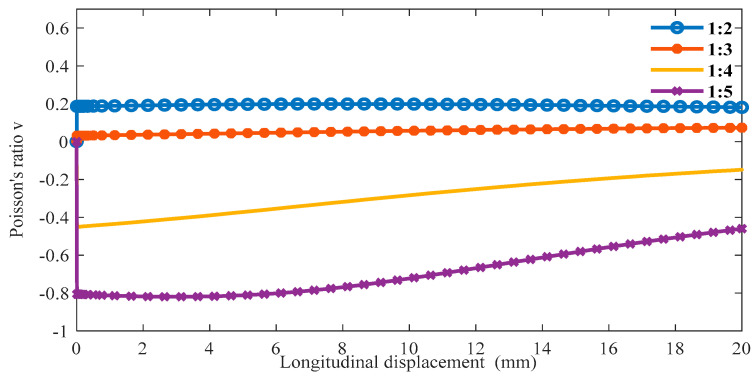
The relationship between longitudinal displacements and Poisson’s ratio v under different β (α = 60°).

The boundary conditions are the same as in the previous section in ABAQUS, and we can obtain the following data in Figure 14 and Figure 15. The trend of lateral and longitudinal displacements is similar to that of α = 45°, but the lateral displacement is smaller when the longitudinal displacement is the same. The difference between positive and negative values of the Poisson’s ratio is more than that at α = 45°. When β is less than or equal to 1:3, the Poisson’s ratio of the structure can reach 0 and 0.2; when β is less than or equal to 1:4, the Poisson’s ratio of the structure can reach 0.4 and 0.8.

**Figure 16:**
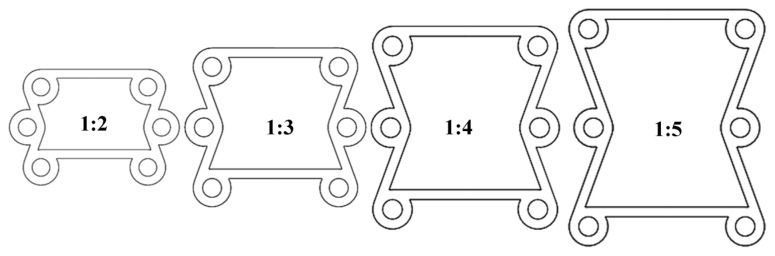
Keeping α = 70° and changing β to 1:2–1:5.

When α=70°, the ratio β between the radius of the ring and the ligament is set to 1:2, 1:3, 1:4, and, 1:5, as shown in Figure 16.

**Figure 17:**
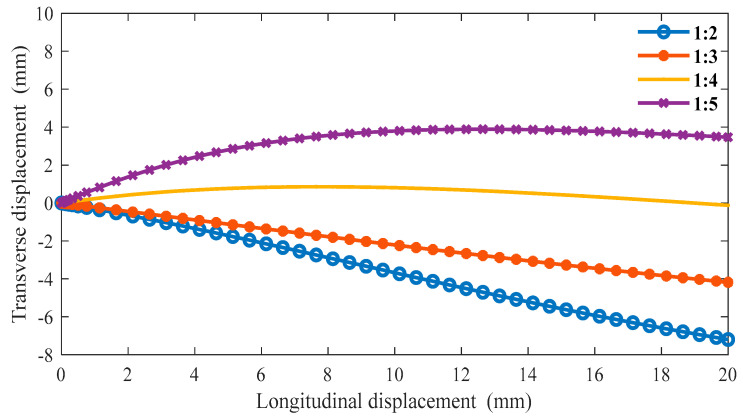
The relationship between transverse and longitudinal displacements under different β (α = 70°).

**Figure 18:**
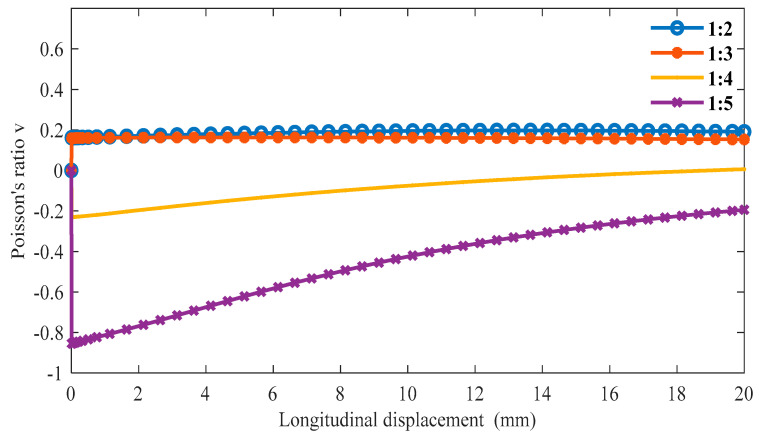
The relationship between longitudinal displacements and Poisson’s ratio v under different β (α = 70°).

The boundary conditions are the same as in the previous section in ABAQUS, and we can obtain the following data. Combining the two previous datasets for α and Figure 17 and comparing to α = 45°and α = 60°, the lateral displacement becomes smaller under the same longitudinal displacement. In addition, as α gradually increases, the effect of the longitudinal displacement on the lateral displacement becomes smaller. In Figure 18, when β is less than or equal to 1:3, the Poisson’s ratio of the structure can reach 0.2, and the Poisson’s ratios for β = 1:2 and β = 1:3 are almost the same. Furthermore, when β = 1:5, the Poisson’s ratio showed an upward trend with the increase of longitudinal displacement. This occurs because the cell has reached its maximum expansion and the distortion of the ring has reached its maximum.

**Figure 19:**
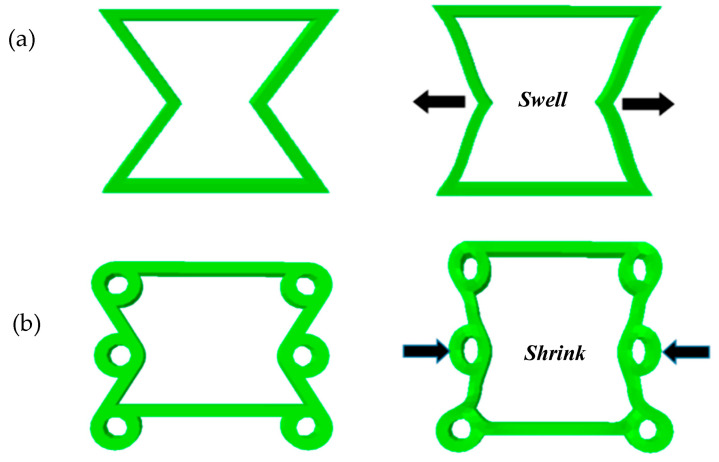
(**a**) The ordinary concave structure is stretched. (**b**) The new structure is stretched.

From the results of the fixed circle radius, it can be observed that when β=1:4, the larger the α, the larger the Poisson’s ratio *v*. In addition, when the α changes from 70° to 75°, the Poisson’s ratio *v* turns from negative to positive under the same load. Furthermore, when angle α is constant, the ratio between the radius of the ring and the ligament changes. Another phenomenon occurs in which Poisson’s ratio *v* becomes larger as β decreases when the same longitudinal displacement is applied. When α=45° and β<1:3, Poisson’s ratio *v* turns from negative to positive under the same load. When α=60° or 70° and β<1:4, Poisson’s ratio *v* goes from negative to positive under the same load. An analysis is conducted based on the above statements. Through the finite element analysis verification, Poisson’s ratio *v* of the structure can turn from positive to negative because of the collapse of the ring when the structure is stretched, as shown in Figure 19.

Because the structure is made of an SMP, the shape memory effect of the SMP can be used to realize the simple deformation of the structure. As described above, Poisson’s ratio of the structure can be changed from positive to negative by simply changing the angle α or the ratio β. Compared with the change of α, changing β is more convenient and can achieve better results. Therefore, we combine the above analysis and integrated the bidirectional deformation principle in Section 3 to design a bidirectional deformation composite structure with variable positive and negative Poisson’s ratio *v*.

## 5. Design and Simulation of Autonomous Variable Structures with Positive and Negative Poisson’s Ratio Based on SMP

Based on the above analysis, we can use ABAQUS to simulate the conversion process of the Poisson’s ratio of the cell from positive to negative. In this section, two types of scaffolds are designed to assist the new type of cells made of shape memory polymers to undergo autonomous changes under the action of temperature fields. In addition, when the scaffold-assisted cell changes, the original changes of the cell are not broken.

### 5.1. Modeling of Elastomer Scaffolds

**Figure 20:**
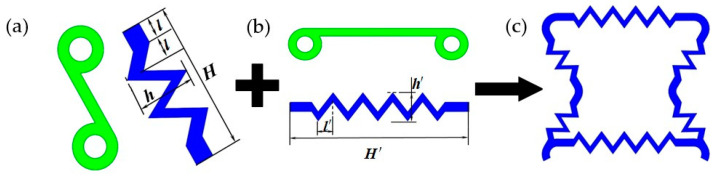
Scaffold A: (**a**) Elastic scaffold of oblique ligaments. (**b**) Elastic scaffold of transverse ligament. (**c**) Elastic scaffold to assist in changing α.

**Figure 21:**
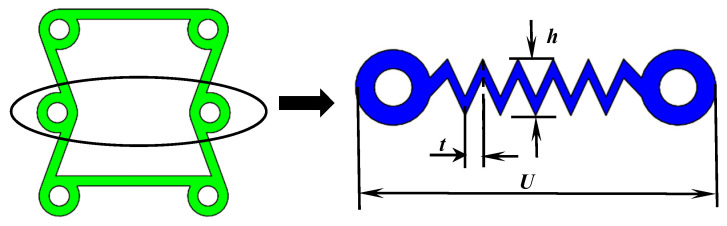
Scaffold A: Elastic scaffold to assist in changing β.

We define the two types of scaffolds as scaffold A and scaffold B, as shown in Figure 20 and Figure 21. In Figure 20a showing scaffold A, the structural parameters of the scaffold bonded to the lateral ligament are h=3(R-r) and H=a. In Figure 20b, the structural parameters of the scaffold bonded to the ligaments on the upper and lower sides are h′=3(R−r), and, where H′ is the length of the ligaments between the upper and lower rings, l′=H′/12. In Figure 21, scaffold B controls α by changing the middle two circles on the cell, and the structural parameters of the scaffold are l″=(U−2R)/12 and h″=3(R−r).

### 5.2. Deformation Process

**Figure 22:**
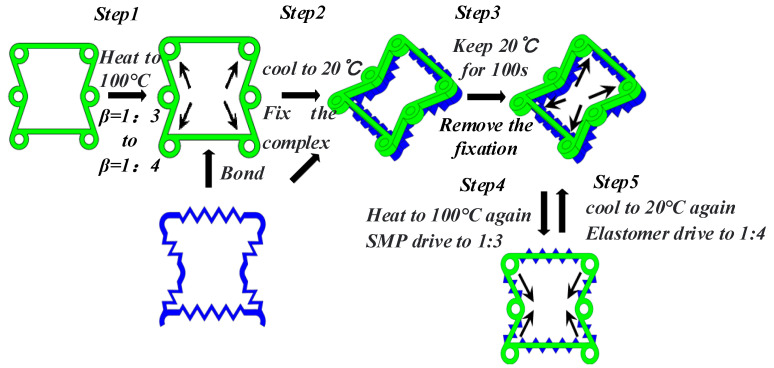
Bidirectional deformation process of composite structure (changing β).

**Figure 23:**
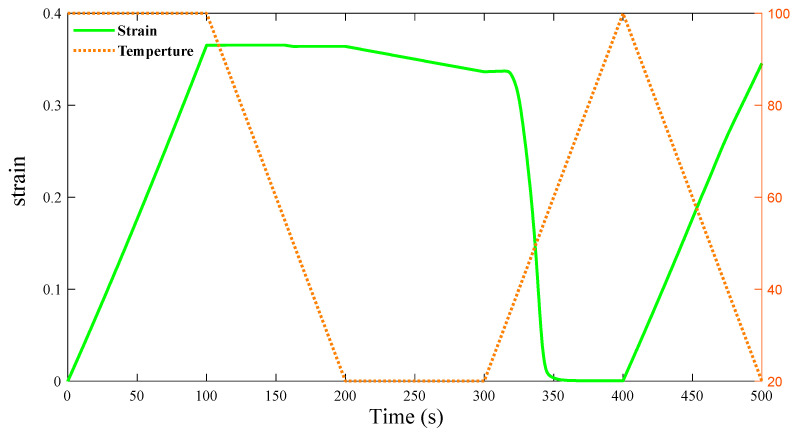
Strain of SMP structure during the whole process, from β = 1:3 to β = 1:4.

The process of automatic adjustment assisted by elastic scaffold A is shown in Figure 22. The process is divided into five steps. Step one: Preheat the cell structure with α=60° and a ratio of 1:3 between the ring and the ligament to 100 °C. When the material reaches a rubbery state, apply a load. This load changes the ratio β between the ligament and the ring to 1:4. Bond the elastomer to the deformed cell while completely immobilizing the formed complex. Step two: With continuous fixation, reduce the temperature to 20 °C which the cooling rate is 0.8 °C/s for 100 s. Step three: Keep the temperature constant for 100 s. Step four: Remove the fixation of the complex, and reheat the material to 100 °C at a rate of 0.8 °C/s for 100 s. In this process, the shape memory polymer produces a shape memory effect that drives the entire complex back to β=1:3, and a prestress appears in the elastic scaffold. Step five: Cool the whole complex to 20 °C again at a cooling rate of 0.8 °C/s for 100 s. In this process, because the elastomer contains a prestress, it is going to drive the whole complex back to the original state, which is a 1:4 ratio between the ring and the ligament. Finally, repeatedly heating the complex to 100 °C or cooling it to 20 °C can achieve autonomous adjustment of the cell Poisson’s ratio from positive to negative. The temperature changes and cell strain of the shape memory polymer are shown in Figure 23.

**Figure 24:**
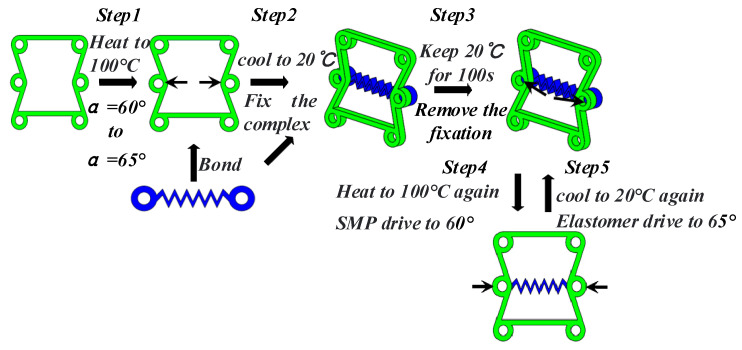
Bidirectional deformation process of composite structure (change α).

**Figure 25:**
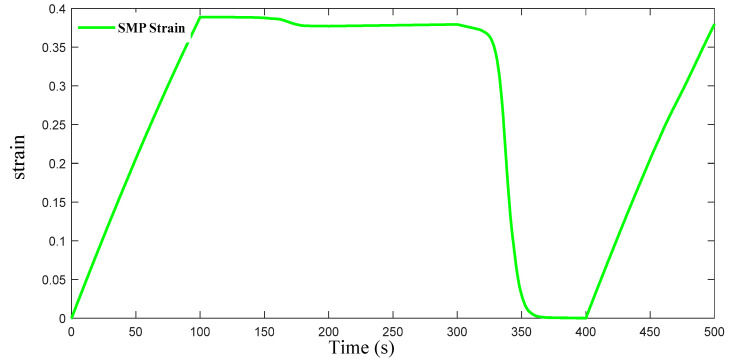
Strain of SMP structure during the whole process, from α = 60° to α = 65°.

The process of automatic adjustment is assisted by elastic scaffold B, as shown in Figure 24. Step one: Preheat cells made of shape memory polymers to 100 °C. When the material reaches a rubbery state, apply a load. This load changes the ratio angle α = 65°. Bond the elastomer to the deformed cell while completely immobilizing the formed complex. Step two: With continuous fixation, reduce the temperature to 20 °C at a cooling rate of 0.8 °C/s for 100 s. Step three: Keep the temperature constant for 100 s. Step four: Remove the fixation of the complex, and reheat the material to 100 °C at a rate of 0.8 °C/s for 100s. In this process, the shape memory polymer produces a shape memory effect that drives the angle back to 60°, and a prestress appears in the elastic scaffold. Step five: Cool the whole complex to 20 °C again at a cooling rate of 0.8 °C/s for 100 s. In this process, because the elastomer contains a prestress and shape memory polymers have no shape memory effect, the elastomer drives the whole complex back to the state in which the angle is 60°. Finally, repeatedly heating the complex to 100 °C or cooling it to 20 °C can achieve autonomous adjustment of the cell Poisson’s ratio from positive to negative. The temperature changes and cell strain of the shape memory polymer are shown in Figure 25.

In Figure 15 and Figure 24, whether α = 60° to α = 65° and whether β = 1:3 to β = 1:4, the maximum strain value is 0.4, the minimum strain value is 0, and the strain trend is the same. When α increases from 60° to 65°, the Poisson’s ratio of the SMP structure changes by about 0.2. When β changes from 1:3 to 1:4, the Poisson’s ratio of the SMP structure changes by about 0.4. In the bidirectional memory deformation of composite structures, it is very necessary to achieve a larger change in Poisson’s ratio by a small strain. Obviously, it is better to change Poisson’s ratio by changing β than changing α when the composite structure deforms autonomously via temperature stimulation.

## 6. Conclusions

In this article, a programmable composite structure with bidirectional mnemonic effects is designed. The viscoelastic constitutive properties of shape memory polymers in ABAQUS are described, and the theory of bidirectional deformation of shape memory polymers is described. We define two structural parameters, α and β. Then, we simulate the changes between longitudinal displacement and transverse displacement, as well as longitudinal displacement and Poisson’s ratio, of the new structure when α and β are changed, respectively. Additionally, the rules of the changes in performance parameters caused by α and β are summarized. We found that the stretching swell effect is gradually transformed into the stretching shrink effect because of the collapse of the ring under the same load, and with the continuous change of structural parameters, the Poisson’s ratio of new structural gradually changes from negative to positive. Finally, two types of elastic scaffolds for auxiliary changes are designed. The programming process of the composite structure is shown, and we simulate two bidirectional deformation programming processes with the assistance of elastic scaffolds in ABAQUS. It is found that the effect of changing β is better than that of changing α during the bidirectional deformation of the composite structure.

One of the key points of metamaterial research is to combine metamaterial and smart material to give the metamaterial corresponding perception ability to the external environment and flexible programmability. Shape memory materials can be well combined with metamaterials, and metamaterials can have programmability by blending with these materials. By combining the deformation principle of shape memory polymer bidirectional deformation, the result not only has the super high recovery rate of shape memory polymers but also has the bidirectional memory effect of shape memory alloys, and the deformation of asymmetric multi-mode can be achieved by this method. The method of combining metamaterials and shape memory materials has great development potential in this article. With the continuous update and development of materials and new structures, the range of changes in Poisson’s ratio and the flexibility of structural changes will also be greatly improved. The article provides a better idea for the combination of new smart materials and metamaterials.

## Data Availability

This manuscript has associated data in a data repository. All data included in this manuscript are available upon request by contacting the corresponding author.

## Appendix A

**Table A1:** The material parameters of shape memory polymers in ABAQUS [23].

Epoxy-Based Shape Memory Polymer		
Expansion: 5.7 × 10^−5^, 50 2.443 × 10^−6^, 55		
Hyperelastic, neo-Hooke:		
393.964, 0.0006452		
Viscoelastic, time = PRONY:		
0.187327	0	3.031 × 10^−5^
0.222863	0	0.0001721
0.257003	0	0.0009768
0.225274	0	0.005545
0.0863276	0	0.03147
0.0144556	0	0.1787
0.00287336	0	1.014
0.00103207	0	5.757
0.000510199	0	32.68
0.000223371	0	185.5
6.41683 × 10^−5^	0	1053
1.60548 × 10^−5^	0	5977
Trs 50, 10.17, 47.35		
